# Topical Analgesic Containing Methyl Salicylate and L-Menthol Accelerates Heat Loss During Skin Cooling for Exercise-Induced Hyperthermia

**DOI:** 10.3389/fphys.2022.945969

**Published:** 2022-07-13

**Authors:** Gang Wang, Tingran Zhang, Anjie Wang, Chansol Hurr

**Affiliations:** ^1^ Integrative Exercise Physiology Laboratory, Department of Physical Education, Jeonbuk National University, Jeonju, South Korea; ^2^ Department of Physical Education, Xinyang Normal University, Xingang, China

**Keywords:** hyperthermia, skin cooling, core temperature, cutaneous vasodilation, topical analgesics

## Abstract

Hyperthermia impairs physical performance and, when prolonged, results in heat stroke or other illnesses. While extensive research has investigated the effectiveness of various cooling strategies, including cold water immersion and ice-suit, there has been little work focused on overcoming the cutaneous vasoconstriction response to external cold stimulation, which can reduce the effectiveness of these treatments. Over-the-counter (OTC) topical analgesics have been utilized for the treatment of muscle pain for decades; however, to date no research has examined the possibility of taking advantage of their vasodilatory functions in the context of skin cooling. We tested whether an OTC analgesic cream containing 20% methyl salicylate and 6% L-menthol, known cutaneous vasodilators, applied to the skin during skin cooling accelerates heat loss in exercise-induced hyperthermia. Firstly, we found that cutaneous application of OTC topical analgesic cream can attenuate cold-induced vasoconstriction and enhance heat loss during local skin cooling. We also revealed that core body heat loss, as measured by an ingestible telemetry sensor, could be accelerated by cutaneous application of analgesic cream during ice-suit cooling in exercise-induced hyperthermia. A blunted blood pressure response was observed during cooling with the analgesic cream application. Given the safety profile and affordability of topical cutaneous analgesics containing vasodilatory agents, our results suggest that they can be an effective and practical tool for enhancing the cooling effects of skin cooling for hyperthermia.

## 1 Introduction

Hyperthermia, a condition marked by an abnormally high body temperature, impairs various physiological functions, reduces physical performance, and heightens the risk of related illnesses such as heat stroke. Individuals who perform intense physical activity in a hot environment, including those who exercise in summer heat, members of the military, and firefighters, are at increased risk for hyperthermia (Douma et al., 2020). To alleviate a hyperthermic state, extensive prior research has focused various cooling strategies including cold water immersion (CWI) (Armstrong et al., 1996; Proulx et al., 2003; Luhring et al., 2016), topical body cooling (Scheadler et al., 2013; Adams et al., 2016; Cuttell et al., 2016), cooling garments (Eijsvogels et al., 2014; Abdallah et al., 2015; Xu et al., 2021), or ice-sheets (Butts et al., 2017), etc. Among those, CWI is considered the current gold standard for treating hyperthermia by most professionals and organizations (Casa et al., 2007; Zhang et al., 2015; Nye et al., 2016). CWI, however, is not always available, as neither whirlpools nor tubs are feasible to maintain in remote locations or sports fields.

Ice-suit, also referred to as cooling garment, are often utilized in sports that are played in hot environments. However, the data suggests that ice-suits have an unacceptable cooling rate (0.03–0.053°C/min) and are not well-suited to the treatment of serious hyperthermic conditions like exertional heat stroke (>40°C) (Keen and Miller, 2017). Although acceptable rates of cooling are generally considered to be >0.08°C/min with an ideal cooling rate being >0.16°C/min (McDermott et al., 2009), the ice-suit is still a useful tool in sports or other outdoor physical activities due to its portability (Brade et al., 2010; Gao et al., 2011; Keen and Miller, 2017). Accordingly, further research for improving the cooling effect of the portable ice-suit for treating hyperthermia is warranted.

External cold stimulation causes vasoconstriction in the cutaneous microvasculature. This response helps maintain a stable core body temperature as the amount of heat transferred from the skin to the core body is decreased due to a substantial reduction in cutaneous blood flow (Charkoudian, 2010; Tansey and Johnson, 2015). In a hyperthermic condition, however, the cutaneous vasoconstriction response to external cold can be a restriction factor because any cooling effect is partly limited to the local region that is directly cooled *via* conduction. In this line, a cooling effect of temperate-water immersion at 26°C on hyperthermic body is comparable to that of CWI at 14°C due to the maintenance of a greater peripheral blood flow during the temperate-water immersion (Taylor et al., 2008).

Topical analgesics, or pain relievers, have been widely utilized for decades by those who participate in sports and other physical activities for pain relief in local muscles and joints. The two primary ingredients in over-the-counter (OTC) topical analgesic products are methyl salicylate (12%–30%) and L-menthol (1%–10%) (Martin et al., 2004; Higashi et al., 2010). Interestingly, previous studies have revealed that methyl salicylate and L-menthol act as cutaneous vasodilators when applied to the skin (Green and Flammer, 1989; Dölen et al., 2015; Craighead and Alexander, 2016; Craighead et al., 2017). These two ingredients are also known to have a synergistic effect in terms of absorption efficacy in the skin, such that methyl salicylate is more absorbed by the skin when in the presence of L-menthol (Yano et al., 1991).

The purpose of the present investigation was to determine whether the application of an OTC topical analgesic cream containing L-menthol and methyl salicylate could augment heat loss during ice-suit cooling in response to hyperthermia. We performed two experiments. In the first, we tested whether cutaneous application of a topical analgesic cream attenuated cold-induced cutaneous vasoconstriction and increased heat loss in the skin during local cooling relative to a control cooling. We then tested whether cutaneous application of topical analgesic cream accelerated a reduction in core body temperature during ice-suit cooling following exercise-induced hyperthermia.

## 2 Materials and Methods

### 2.1 Ethical Approval and Participants

The Institutional Review Board (IRB) at Jeonbuk National University approved all study procedures used in the current experiment (IRB #: JBNU 2020-08-021-001). Participants were given a verbal description of all procedures and informed of the purpose of and risks involved in the study. Written consent was obtained before the study began. The study conformed to the provisions of the Declaration of Helsinki.

Ten healthy men (Study 1: age 27.30 ± 2.63 years, height 180.30 ± 7.42 cm, body weight 74.12 ± 7.39 kg, BMI 22.75 ± 0.89 kg·m^−2^, body fat 13.73% ± 1.13%; Study 2: age 28.20 ± 2.62 years, height 178.20 ± 5.85 cm, body weight 73.32 ± 5.71 kg, BMI 23.07 ± 0.83 kg·m^−2^, body fat 13.57 ± 1.03%) participated in the current study. Participants were excluded if they were smokers, had an allergy to cold stimulation or topical analgesic cream, a history of any cardiovascular, respiratory, metabolic diseases, or any existing symptoms of pain. Participants were instructed to maintain their regular diet during the participation period in Study 1 but were asked to be fasted overnight (∼12 h) due to removal of the artificial temperature deviation of ingestible temperature sensors (Domitrovich et al., 2010). Also, they were asked to refrain from strenuous exercise, alcohol, and caffeine consumption during the 24 h preceding the visit. During the first visit, after completion of a written consent form, participants were familiarized with the experimental procedures. Body mass (kg) and body fat (%) were measured during the first visit (InBody 720, InBody Co., Seoul, South Korea).

### 2.2 Experimental Procedures and Measurement

#### 2.2.1 (Study 1) Procedures and Measurement

**FIGURE 1:**
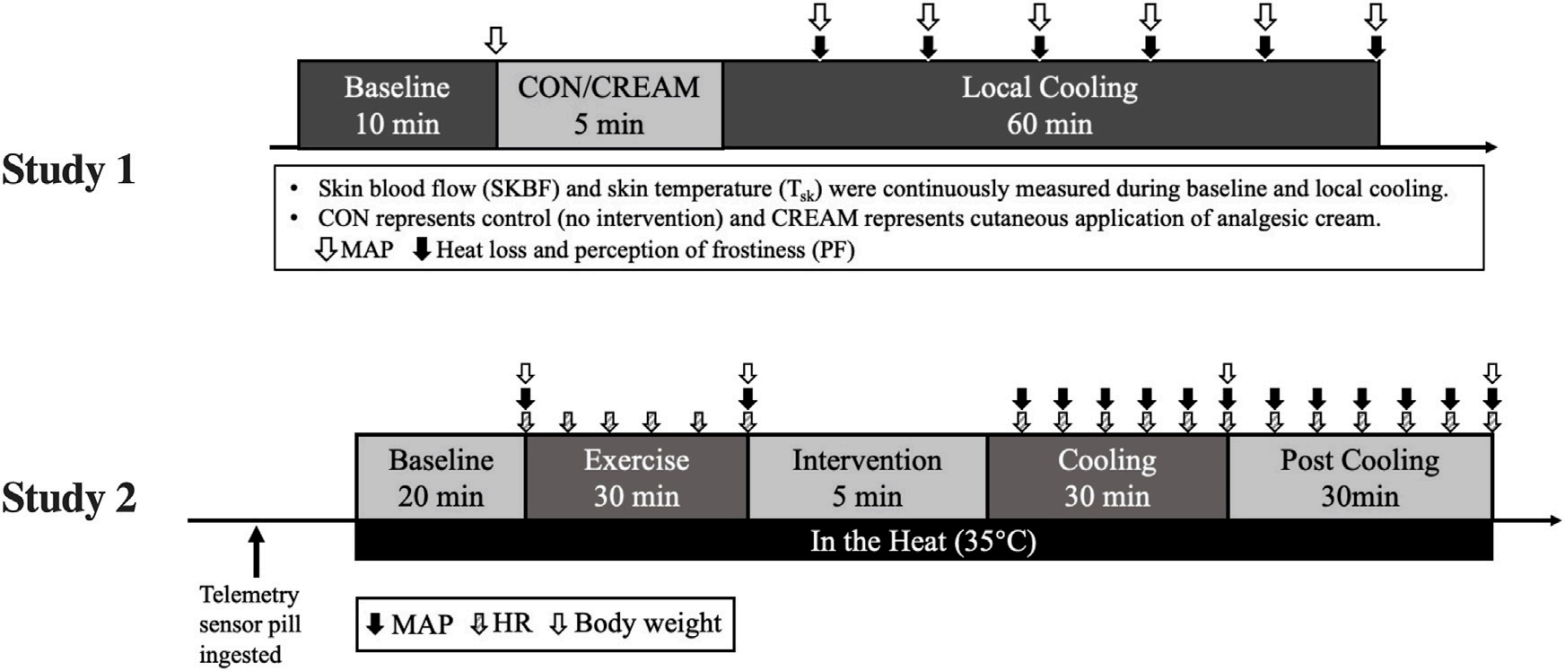
The experimental design. MAP, mean arterial pressure; HR, heart rate; CON, control cooling; CREAM, cooling with cutaneous application of analgesic cream.

A schematic of our experimental protocol is shown in Figure 1. The experiment was conducted in a temperature-controlled laboratory (∼25°C and 50% humidity). After a familiarization visit, each participant visited the laboratory twice, whereupon they were once subjected to a control cooling without any intervention (CON) and once subjected to an experimental cooling with analgesic cream applied to the cooled region of the body (CREAM). The order in which these occurred was randomized with at least 48 h separation between them. Upon arrival, participants were asked to rest in a patient bed in a semi-supine position for 10 min. A soft cushion was placed under the left leg to minimize movement during data collection. During this resting period, two laser doppler probes (VP7 A/T with moorVMS-LDF2; Moor Instruments, Wilmington, DE, United States) were placed on the anterior thigh 15 cm placed apart to minimize inter-probe interaction. A line was measured between the anterior superior iliac spine and the superior border of the patella. Then, two spots for laser doppler probes were determined from the center of the line. Following the instrumentation, baseline skin blood flow (SkBF) and skin temperature (T_sk_) were recorded for 10 min using laser doppler probes, followed by three measurements of intermittent blood pressure measurement (HEM-770A; Omron Healthcare, Japan). SkBF and T_sk_ were averaged during the last min of the baseline period. Mean arterial blood pressure (MAP) was calculated as one-third pulse pressure plus diastolic blood pressure and cutaneous vascular conductance (CVC) was calculated as the ratio of SkBF to MAP.

Following the baseline measurement, four drops of topical analgesic cream (0.4 ml) were evenly applied to the left anterior thighs (25 cm × 25 cm) of those in the CREAM group while no cream was applied to those in CON. An experimenter wore latex globes to prevent cream absorption to the hands. In the CREAM group, laser doppler probes were detached for cream application after baseline measurement and reattached to the same spot after the cream was applied. The anterior thigh was then covered with a customized PVC tube-lined water-perfused pad (28 cm × 27 cm). Ice water (∼2°C) in the 40 L heat-insulated container circulated through the pad at a flow rate of 10 ml/min *via* a peristaltic pump. Once the pad was placed, SkBF and T_sk_ were continuously measured for 1 h. For data analysis, a 60-min period was divided into six 10-min cycles and SkBF and T_sk_ were averaged during last 1 min of each cycle. Also, MAP and perception of frostiness (PF) were measured at the end of each cycle using a visual analogue scale (Koehn et al., 2012).

#### 2.2.2 Cutaneous Application of Topical Analgesic Cream

The topical analgesic cream that was used in the current study consisted of 20% methyl salicylate, 6% L-menthol, and other additives including lanolin and mineral oil, sorbitanmonostearate (surfactant), polysorbate 60 (surfactant), trolamine, and purified water (Antiphlamine-S, Yuhan, South Korea). From our pilot data (*n* = 8), we confirmed that 0.64 μl/cm^2^ of cream (i.e., 0.4 ml (four drops) applied to a 25 cm × 25 cm area of skin induced a potent vasodilatory effect in the skin (80%–115% increase relative to resting SkBF) over the course of 1 h with ∼1°C increase in T_sk_ (Table 1).

**TABLE 1:** Skin blood flux (SkBF) and skin temperature (T_sk_) in response to cutaneous application of analgesic cream from pilot study.

Variables		Time (min)						
		Baseline	10	20	30	40	50	60
SkBF	Flux (A.U.)	30.73 ± 5.74	49.99 ± 6.24*	53.55 ± 7.30*	58.63 ± 7.62**	61.40 ± 10.14**	58.53 ± 9.89**	62.21 ± 11.95*
	% baseline (%)	0	79.58 ± 19.44	95.12 ± 23.68	115.30 ± 27.85	112.65 ± 20.92	100.96 ± 17.23	106.85 ± 15.31
T_sk_ (°C)		31.61 ± 0.38	32.39 ± 0.41**	32.45 ± 0.44*	32.51 ± 0.48*	32.61 ± 0.46*	32.69 ± 0.49*	32.66 ± 0.54*

One-way repeated measures ANOVA with a Dunnett post-hoc test for all. Data are presented as the mean ± SD (*n* = 8). *p < 0.05 vs. baseline. **p < 0.01 vs. baseline.

#### 2.2.3 Indirect Measurement of Heat Loss in the Skin

During cooling, water that circulated in the pad was separately collected every 10 min (∼100 ml) in the heat-insulated cup, and water temperature was measured at the end of each 10 min period using a digital thermometer. Since the temperature of the ice water in the 40 L-container prior to circulation through the pad was constant at 2°C, the temperature of the circulated water can serve as an indirect indicator of heat loss in the skin (Table 1).

#### 2.2.4 (Study 2) Procedures and Measurement

Based on our findings in Study 1, we performed a second experiment, the schematic protocol of which is shown in Figure 1. Upon arrival, subjects immediately ingested a telemetry sensor pill (HQ, Palmetto, FL, United States) for measuring core body temperature (T_core_), and drank 200 ml water warmed at 37°C. No additional water was allowed afterwards. T_core_ and heart rate (HR, Polar Electro Inc., Bethpage, NY, United States) was continuously recorded throughout the entire protocol. 2 h after the ingestion of the sensor pill, subjects went into the temperature-controlled laboratory with a temperature maintained at 35°C (∼50% humidity) and all experimental procedures including Baseline, Exercise, Cooling, and Post Cooling were completed in a hot environment (Figure 1). Following the 20-min resting baseline period (Baseline), subjects performed a 30 min high-intensity interval exercise, during which T_core_ increased by 1.78°C ± 0.23°C from the baseline (Baseline 37.04 ± 0.19 vs. Post Exercise 38.82°C ± 0.17°C for all groups combined). Interval exercise consisted of 30-s jumping jacks, high knees, squats, side and reverse lunges, and cycling with 5 s rest given between sets.

Once hyperthermia was induced, three different 30-min interventions—1) rest without cooling (CON), 2) control cooling using an ice-suit (C), and 3) same cooling with analgesic cream applied to cooled regions (C + CREAM)—were administered in a randomized order with a minimum of 1-week between interventions. After these interventions, subjects in C and C + CREAM took off the ice-suit and data acquisition continued for another 30 min (Post Cooling). The analgesic cream was evenly applied to multiple local regions that was cooled by an ice-suit. The amount of analgesic cream per skin area was same as that used in Study 1 (0.64 μl/cm^2^).

Intermittent MAP was measured at the end of Baseline, after Exercise, and at the end of every 5 min period during Cooling and Post Cooling. For sweat loss, nude body mass was measured at the end of Baseline, Exercise, Cooling, and Post Cooling. Also, perception of discomfort (PD) and PF was assessed every 10 min during Cooling and Post Cooling.

#### 2.2.5 Ice-Suit

An ice-suit consists of two parts, a torso and legs. The inner surface of the suit was attached to gel-filled silicone cells with Velcro. The gel was a mixture of purified water with non-toxic chemicals that maintained a temperature of 7°C (Ice Tube, COOLMEDICS Korea, South Korea). The chest, shoulders, abdomen, back, anterior thighs, knees, and calves were cooled. All silicone cells in the suit were replaced with freshly frozen cells after 15 min of Cooling to maintain the cooling effect.

### 2.3 Statistical Analysis

Data are expressed as mean ± SD, and statistical significance was accepted as *p* < 0.05. Variables were analyzed using two-way repeated-measures ANOVA, followed by a Tukey’s post hoc analysis; the factors were cooling conditions (CON/CREAM in Study 1 or CON/C/C + CREAM in Study 2) and time (or stage). Statistical analyses were performed using GraphPad Prism 9.2.0 (GraphPad Software, La Jolla, CA, United States).

## 3 Results

### 3.1 Study 1

#### 3.1.1 Skin Blood Flow and Cutaneous Vascular Conductance

SkBF steadily decreased during the 60-min cooling period in both CON and CREAM (Time Effect: *p* < 0.0001). However, SkBF was significantly elevated at the onset of cooling in CREAM relative to CON (CON 16.22 ± 3.86 vs. CREAM 41.97 ± 18.52 A.U, *p* < 0.0001) and higher SkBF was maintained throughout the cooling (Group Effect: *p* = 0.0002). Similarly, CVC declined in both conditions during cooling (Time Effect: *p* < 0.0001) but was elevated in CREAM relative to CON (Group Effect: *p* = 0.0002). No changes in MAP were observed in either condition during cooling (Time and Group Effects: *p* = 0.551 and 0.763, respectively) (Table 2).

**TABLE 2:** Mean arterial pressure (MAP) and perception of frostiness (PF) in study 1.

Variables	Intervention	Baseline	Cooling time (min)					
			10	20	30	40	50	60
MAP (mmHg)	CON	81.39 ± 8.48	81.09 ± 9.67	81.39 ± 9.69	79.40 ± 11.14	82.04 ± 9.40	81.23 ± 9.95	81.89 ± 8.92
	CREAM	81.62 ± 7.65	80.79 ± 7.49	81.38 ± 7.09	80.05 ± 7.25	80.69 ± 7.09	80.13 ± 7.39	80.48 ± 7.68
PF (0–10)	CON	0	2.40 ± 1.17	2.70 ± 1.06	2.60 ± 0.97	2.90 ± 0.88	2.70 ± 0.82	2.40 ± 0.69
	CREAM	0	2.10 ± 1.19	2.40 ± 1.27	2.30 ± 0.95	2.30 ± 0.82*	2.30 ± 0.95*	2.00 ± 0.82*

CON, control cooling; CREAM, cooling with cutaneous application of analgesic cream; PF, perception of frostiness. Two-way repeated measures ANOVA with a Tukey’s post-hoc test for all. Data are presented as the mean ± SD (*n* = 10). *p < 0.05 vs. CON.

#### 3.1.2 Skin Temperature (T_sk_) and Circulated Water Temperature (Heat Loss)

T_sk_ decreased in both conditions during cooling (Time Effect: *p* < 0.0001), but no differences were found between the CON and CREAM groups (Group Effect: *p* = 0.649) (Figure 3A). In both groups, circulated water temperature was highest in the first 10 min of cooling, and this decreased as cooling progressed (Time Effect: *p* < 0.0001) (Figure 3B). Interestingly, circulated water temperature was higher in CREAM than in CON (Group Effect: *p* < 0.0001) (Figure 3B). Once cooling began, the perception of frostiness (PF) was lower in CREAM between the 40- and 60-min marks than in CON (40 min: CON 2.90 ± 0.88 vs. CREAM 2.30 ± 0.82, *p* = 0.0002; 50 min: CON 2.70 ± 0.82 vs. CREAM 2.30 ± 0.95, *p* = 0.027; 60 min: CON 2.40 ± 0.69 vs. CREAM 2.00 ± 0.82, *p* = 0.027) (Table 2).

### 3.2 Study 2

#### 3.2.1 Core Temperature (T_core_)

T_core_ during a 20-min baseline established in the heat was 36.92°C ± 0.23°C (all groups combined) and no group differences were found (*p* = 0.212) (Figure 4A). During 30 min of exercise, T_core_ increased across all three conditions (Baseline 36.92 ± 0.23 vs. Post Exercise 38.82°C ± 0.17°C, *p* < 0.0001) with no differences observed between the conditions (Baseline CON 37.04 ± 0.16 vs. C 37.04 ± 0.26 vs. C + CREAM 37.03°C ± 0.14°C, *p* = 0.718; Post Exercise CON 38.83 ± 0.16 vs. C 38.82 ± 0.19 vs. C + CREAM 38.81°C ± 0.18°C, *p* = 0.957) (Figure 4A). During the first 30 min of cooling, all three groups showed a decrease in T_core_ (Time Effect: *p* < 0.0001). A more significant decrease in T_core_ was found in C compared to CON (*p* < 0.01 for all time points between 10 and 30 min of Cooling) (Figure 4A). Interestingly, T_core_ was even lower in C + CREAM than in C (*p* < 0.05 for all time points during Cooling), and this lowered T_core_ lasted throughout Post Cooling (*p* < 0.05 for all time points during Post Cooling) (Figure 4A).

Area under the curve (AUC) analysis revealed that T_core_ AUC was lower in C and C + CREAM relative to CON during Cooling (CON 231.65 ± 1.13 vs. C 228.48 ± 1.03 AUC, *p* < 0.0001; CON 231.65 ± 1.13 vs. C + CREAM 227.46 ± 0.81 AUC, *p* < 0.0001). T_core_ AUC was lower in C + CREAM than in C (C 228.48 ± 1.03 vs. C + CREAM 227.46 ± 0.81 AUC, *p* = 0.007) (Figure 4B). A similar pattern in T_core_ AUC emerged after analysis of Post Cooling data (CON 229.07 ± 1.33 vs. C 224.88 ± 0.83 AUC, *p* < 0.0001; CON 229.07 ± 1.33 vs. C + CREAM 224.05 ± 0.93 AUC, *p* < 0.0001; C 224.88 ± 0.83 vs. C + CREAM 224.05 ± 0.93 AUC, *p* = 0.028) (Figure 4B).

We also analyzed the cooling rate during Cooling (Figure 4C). During the first 10 min, the cooling rate was higher in C and C + CREAM than in CON (CON 0.006 ± 0.012 vs. C 0.048°C ± 0.011°C/min, *p* = 0.0002; CON 0.006 ± 0.012 vs. C + CREAM 0.067 ± 0.018°C/min, *p* = 0.0001). The cooling rate was higher in C + CREAM than in C (C 0.048 ± 0.011 vs. C + CREAM 0.067 ± 0.018°C/min, *p* = 0.019) (Figure 4C). Between 10 and 20 min of Cooling, the cooling rate was higher in C than in CON (CON 0.021 ± 0.011 vs. C 0.040 ± 0.008°C/min, *p* = 0.0102). No other differences between the groups were observed (CON 0.021 ± 0.011 vs. C + CREAM 0.034 ± 0.012°C/min, *p* = 0.137; C 0.040±0.008 vs. C + CREAM 0.034 ± 0.012°C/min, *p* = 0.196). Nor, during the final 10 min of Cooling, were any group differences found (CON 0.019 ± 0.014 vs. C 0.020 ± 0.011 vs. C + CREAM 0.027 ± 0.020°C/min, *p* > 0.05 for all comparisons) (Figure 4C).

#### 3.2.2 Mean Arterial Blood Pressure, Heart Rate, and Sweat Loss

All three groups showed a decrease in MAP during Cooling and Post Cooling (Time Effect: *p* < 0.0001) (Figure 5A). However, MAP was higher in C than in CON after 20 min of Cooling (20 min: CON 90.32 ± 1.71 vs. C 93.87 ± 2.16 mmHg, *p* = 0.003; 25 min: CON 89.52 ± 2.89 vs. C 92.95 ± 2.80 mmHg, *p* = 0.0003; 30 min: CON 89.08 ± 2.24 vs. C 92.02 ± 2.00 mmHg, *p* = 0.015). MAP was also higher in C than in C + CREAM after 15 min of Cooling (15 min: C 92.92 ± 2.94 vs. C + CREAM 88.80 ± 1.73 mmHg, *p* = 0.003; 20 min: C 93.87 ± 2.16 vs. C + CREAM 89.87 ± 1.49, *p* = 0.0004; 25 min: C 92.95±2.80 vs. C + CREAM 88.63 ± 1.42 mmHg, *p* = 0.002; 30 min: C 92.02 ± 2.00 vs. C + CREAM 89.02 ± 1.39 mmHg, *p* = 0.007). No difference in MAP between CON and C + CREAM was observed during Cooling (*p* > 0.05 for all time points). Between 20 and 25 min Post Cooling, a higher MAP was observed in C + CREAM than in CON (*p* < 0.05 for both time points).

HR decreased in all three groups after exercise, but C and C + CREAM had a lower HR than CON (*p* < 0.01 for all time points) (Figure 5B). At the 5 and 15 min marks of the Cooling recovery period, HR was lower in C + CREAM than in C (5 min: C 108.40 ± 7.59 vs. C + CREAM 100.00 ± 10.34 beats/min, *p* = 0.0049; 15 min: C 98.90 ± 6.64 vs. C + CREAM 93.20 ± 9.64 beats/min, *p* = 0.039).

Accumulative sweat loss increased during exercise in all three groups (Figure 5C). During Cooling, C showed lower sweat loss than CON (CON 1.72 ± 0.19 vs. C 1.53 ± 0.22 kg, *p* = 0.003) while no significant difference was found between CON and C + CREAM (CON 1.72 ± 0.19 vs. C + CREAM 1.52 ± 0.29 kg, *p* = 0.069). During Post Cooling, both C and C + CREAM showed less accumulative sweat loss than CON (CON 2.08 ± 0.27 vs. C 1.65 ± 0.10 kg, *p* = 0.001; CON 2.08 ± 0.27 vs. C + CREAM 1.64 ± 0.34, *p* = 0.021). We observed no differences in sweat loss between C and C + CREAM during Cooling and Post Cooling (*p* > 0.05 for all).

#### 3.2.3 Perception of Discomfort and Perception of Frostiness

Perception of discomfort (PD) was similar between groups at the onset of intervention (CON 7.60 ± 0.84 vs. C 7.40 ± 0.69 vs. C + CREAM 7.2 ± 1.39, *p* > 0.05 for all comparisons) (Table 3). Overall, C and C + CREAM participants reported lower PD than those in CON. At the 20 min mark of Cooling as well as the 10 and 20 min marks of Post Cooling, PD was lower in C + CREAM relative to C (Table 3). Perception of frostiness (PF) decreased during the 30 min Cooling and Post Cooling periods in both C and C + CREAM (Time Effect: *p* < 0.0001). PF in both C and C + CREAM showed increases at the 20 min mark of Cooling due to the replacement of the ice gels. No differences were found between these groups (*p* = 0.117) (Table 3).

**TABLE 3:** Perception of discomfort (PD) and perception of frostiness (PF) during cooling and post cooling in study 2.

Variables	Intervention	Onset of cooling	Cooling (min)			Post cooling (min)		
			10	20	30	40	50	60
PD	CON	7.60 ± 0.84	6.50 ± 0.53	5.30 ± 0.48	4.80 ± 0.63	4.40 ± 0.52	4.10 ± 0.74	4.00 ± 0.82
	C	7.40 ± 0.69	4.50 ± 1.84*	5.30 ± 2.06	3.10 ± 1.19*	2.40 ± .35*	2.00 ± 1.41*	1.50 ± 1.65*
	C + CREAM	7.20 ± 1.39	2.40 ± 1.58*	3.80 ± 1.55*^#^	1.60 ± 1.26*	0.50 ± 1.08*^#^	0.50 ± 0.85*^#^	0.50 ± 0.85*
PF	C	6.50 ± 1.18	3.50 ± 1.51	5.60 ± 1.96	2.20 ± 1.32	0.70 ± 0.82	0.10 ± 0.32	0
	C + CREAM	5.80 ± 1.93	2.50 ± 2.22	4.80 ± 1.48	1.20 ± 1.39	0.10 ± 0.32	0	0

CON, rest recovery with no intervention; C, control cooling; C + CREAM, cooling with cutaneous application of analgesic cream. Two-way repeated measures ANOVA with a Tukey’s post-hoc test for all. Data are presented as the mean ± SD (*n* = 10). *p < 0.05 vs. CON. ^#^ *p* < 0.05 vs. C.

## 4 Discussion

**FIGURE 2:**
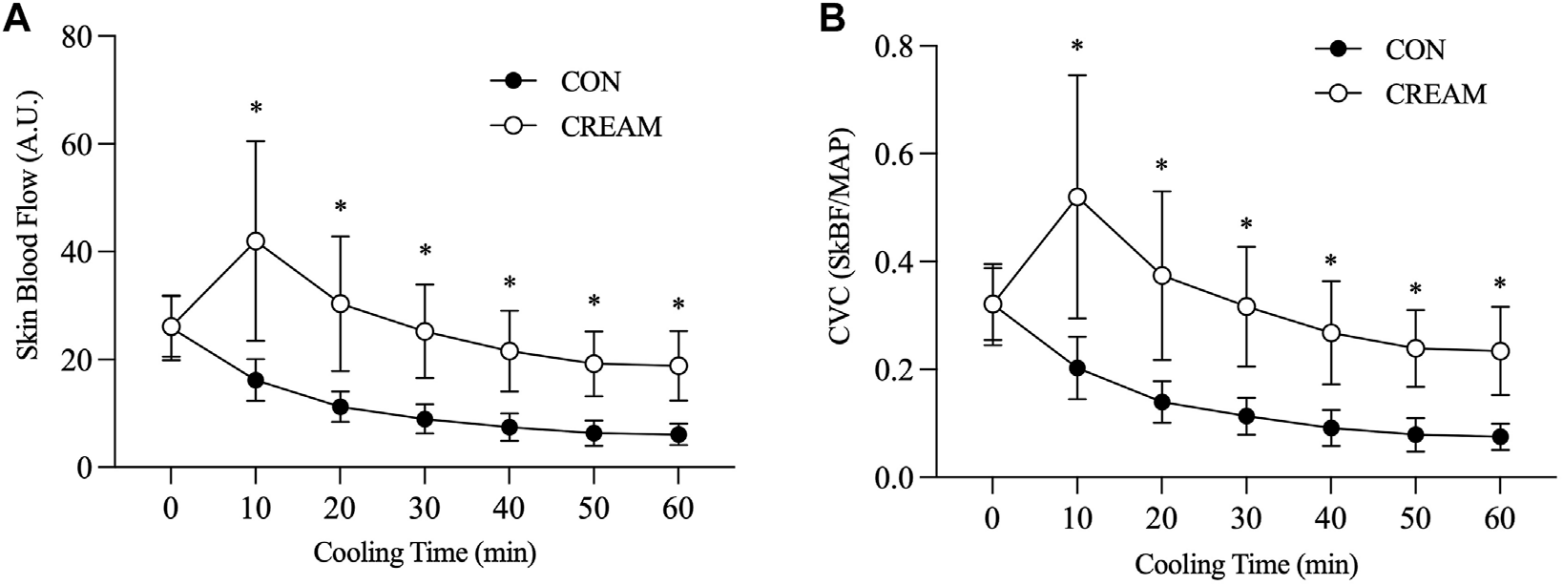
Skin blood flow (SkBF) and cutaneous vascular conductance (CVC) during local skin cooling. SkBF **(A)** and CVC **(B)** during Baseline (0 min) and 60-min local cooling are shown. CON, control cooling; CREAM, cooling with cutaneous application of analgesic cream. Two-way repeated measures ANOVA with a Tukey’s post-hoc test for all. Data are presented as the mean ± SD (*n* = 10). *p < 0.05 vs. CON.

**FIGURE 3:**
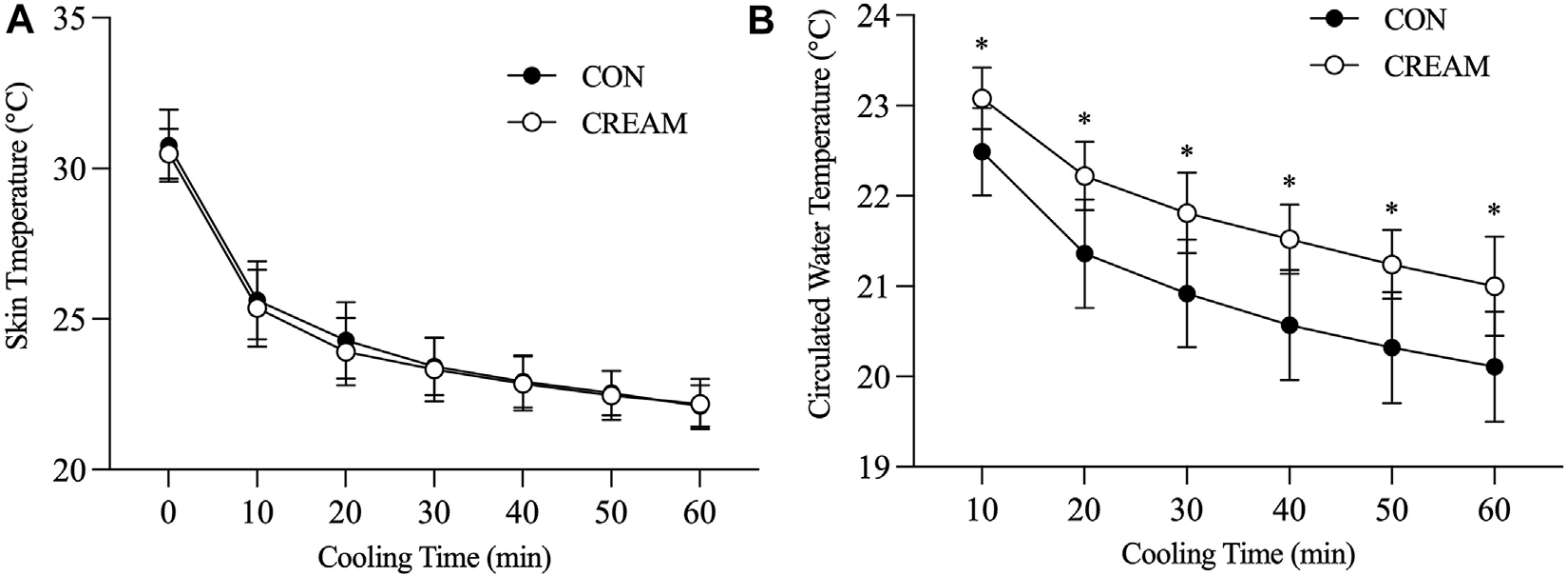
Skin temperature (T_sk_) and circulated water temperature during local skin cooling. T_sk_ **(A)** and circulated water temperature **(B)** during Baseline (0) and 60-min local cooling are shown. CON, control cooling; CREAM, cooling with cutaneous application of analgesic cream. Two-way repeated measures ANOVA with a Tukey’s post-hoc test for all. Data are presented as the mean ± SD (*n* = 10). *p < 0.05 vs. CON.

We investigated the effect of OTC analgesic cream containing 20% methyl salicylate and 6% L-menthol on heat loss during ice-suit cooling in exercise-induced hyperthermia. The analgesic cream acted as a vasodilator in the skin for 1 h (80%–115% from baseline) with a slight but observable increase in T_sk_ (Table 1). In Study 1, we showed that cutaneous application of OTC topical analgesic cream attenuated cold-induced vasoconstriction and enhanced heat loss (i.e., circulated water temperature) in the skin during local cooling, while T_sk_ similarly declined in all conditions during local skin cooling (Figures 2, 3).

**FIGURE 4:**
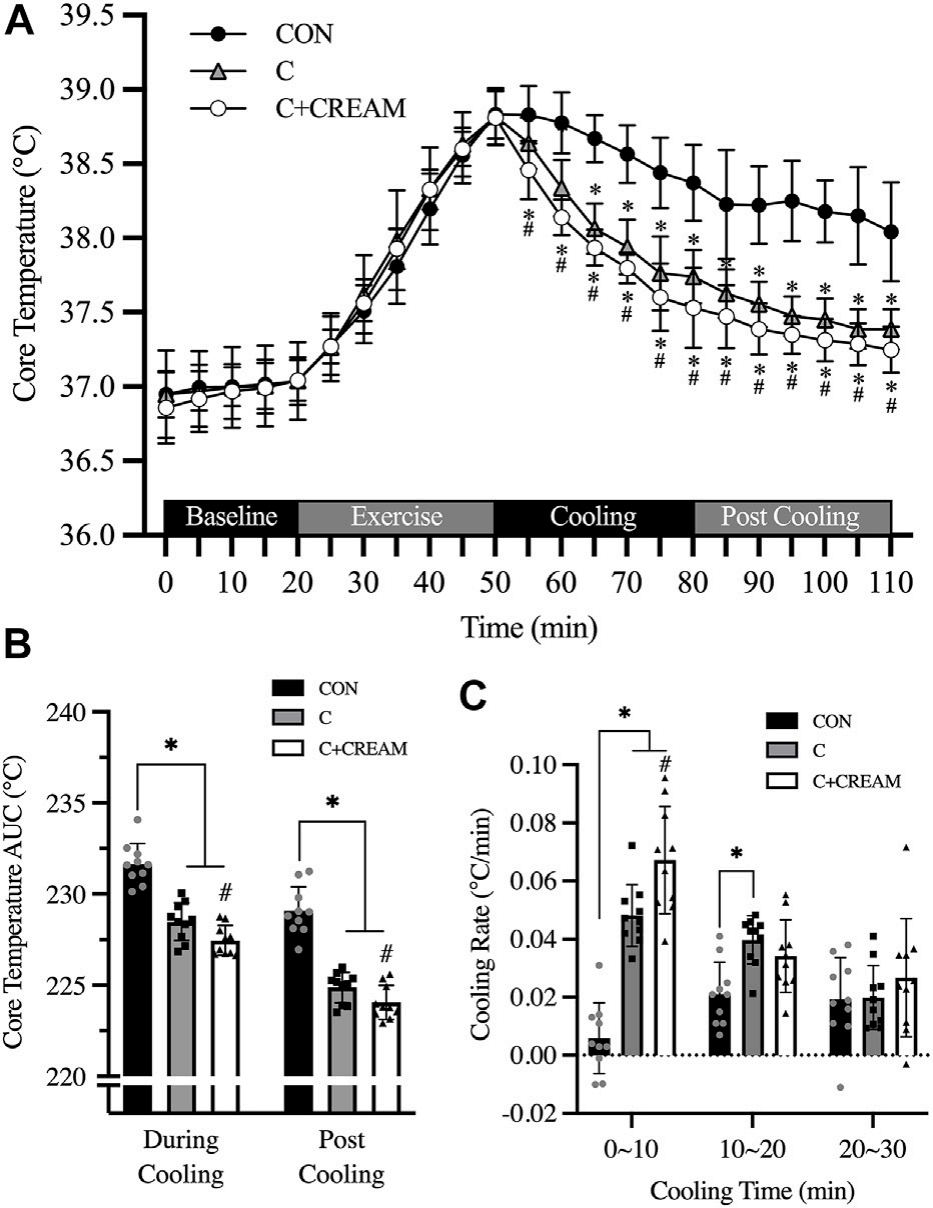
Changes in core temperature (T_core_). Changes in T_core_ during baseline, exercise, cooling, and post cooling in study 2, recorded by ingestible telemetry sensor, are presented **(A)**. Area under the curve (AUC) for T_core_ during cooling and post cooling **(B)** and cooling rates during cooling **(C)** are presented. CON, control cooling; C, ice-suit cooling; C + CREAM, ice-suit cooling with cutaneous application of analgesic cream. Two-way repeated measures ANOVA with a Tukey’s post-hoc test for all. Data are presented as the mean ± SD (*n* = 10). *p < 0.05 vs. CON. ^*#*^ *p* < 0.05 vs. C.

Based on our findings in Study 1, we performed an additional experiment and determined that cutaneous application of analgesic cream during ice-suit cooling accelerated core body heat loss in exercise-induced hyperthermia (Figure 4). In sum, the increased cooling effect of analgesic cream applied during skin cooling on the body’s core appears to be the result of cream-induced cutaneous vasodilation that partially counteracts cold-induced vasoconstriction, thereby increasing heat transfer. However, it should be noted that SkBF and CVC were not directly measured during whole-body cooling in Study 2. Thus, the observed effect is only speculative.

Local cooling leads to vasoconstriction through a number of different mechanisms, including norepinephrine (NE) synthesis, NE release, adrenergic receptors, nitric oxide (NO), etc. (Alba et al., 2019). One prior study provides evidence that activation of the Rho-kinase (ROCK) signaling mechanism is involved in cold-induced vasoconstriction (Lang et al., 2009). In our experiment, cold-induced vasoconstriction in the cutaneous microvasculature was substantially attenuated in the CREAM group in Study 1, such that skin blood flow and conductance were almost twice as high as it had been during the control cooling (Figures 2A,B). Taking into account the main components in our analgesic, we speculate that vasodilatory actions of L-menthol and methyl salicylate played a pivotal role in blunting cold-induced vasoconstriction during skin cooling.

L-menthol is a cutaneous vasodilator that operates *via* various mechanisms including eNOS, L-type voltage gated calcium blockade, EDHF, etc. [see review paper by Silva (Silva, 2020)]. Interestingly, menthol is also known to suppress the RhoA/ROCK pathway (Nunes et al., 2010; Fernández-Tenorio et al., 2011; Xiong et al., 2017), which is one of the mechanisms implicated in the process of cold-induced vasoconstriction. Along with L-menthol’s other vasodilatory actions, suppression of the RhoA/ROCK pathway by L-menthol may have blunted the cold-induced RhoA/ROCK activation that leads to translocation of the α_2C_-adrenergic receptor (Chotani et al., 2000; Bailey et al., 2004).

Methyl salicylate is a naturally occurring compound widely used in nonsteroidal anti-inflammatory drugs (NSAIDs) for the treatment of muscle and joint pain. Ohta et al. (2009) showed that methyl salicylate is an effective agonist for transient receptor potential vanilloid subtype 1 (TRPV1), which is involved in sensory nerve nocicepteive signaling. TRPV1 plays a role in thermogenic regulation, such that inhibition of TRPV1 activates heat gain mechanisms, including cutaneous vasoconstriction, *via* the elevated sympathetic drive (Alawi et al., 2015). Cold stimulation suppresses TRPV1 activation (Chung and Wang, 2011). Read together, these studies suggest that the application of methyl salicylate during cooling may activate TRPV1, which is concomitantly suppressed by cold stimulation, thereby resulting in a blunting of cold-induced vasoconstriction *via* the attenuated sympathetic drive. This has not been confirmed, however, and future investigations will have to determine the molecular mechanisms underlying the phenomenon of blunted cold-induced vasoconstriction by cutaneous application of analgesic cream.

Previous data suggests that cutaneous application of menthol evokes heat-conservation responses, including vasoconstriction. Recent studies by Kounalakis and Botonis showed that whole-body application of menthol results in a slower reduction in rectal temperature during CWI, possibly due to a sympathetic-mediated thermoregulatory mechanism (Kounalakis et al., 2010; Botonis et al., 2018). The cutaneous analgesic product used in the current study contained of large amount of methyl salicylate (20%) in addition to L-menthol (6%), which makes a direct comparison with this and other prior works is difficult (Kounalakis et al., 2010; Botonis et al., 2018), as methyl salicylate is known to be involved in the inhibition of sensory nerves and perception (Ohta et al., 2009). Also, analgesic cream was applied only to a small area. Whole-body application during CWI may initiate greater sympathetic-mediated heat-conservation responses. Previous work has confirmed that topical menthol application decreases temperature in muscle and skin, which may be explained by menthol-induced vasoconstriction (Lasanen et al., 2016; Hunter et al., 2018). Alternatively, the lowered temperatures observed may have been due to evaporative heat loss caused by ethanol rather than menthol action *per se* (Hunter et al., 2018). Menthol increases blood perfusion in the provoked skin region, and also induces vasoconstriction in non-provoked vascular beds (Silva, 2020). As we did not assess the caliber of deep arteries, it is unclear whether vasoconstriction occurred there during skin cooling with analgesic cream. It is clear that blood perfusion in the skin, as assessed by laser doppler flowmetry, exhibited a 80%–115% increase from baseline caused by the application of the analgesic cream alone (Table 1), which is indicative of potent vasodilation in the skin. However, explanations for this discrepancy remain equivocal, and additional studies are warranted.

**FIGURE 5:**
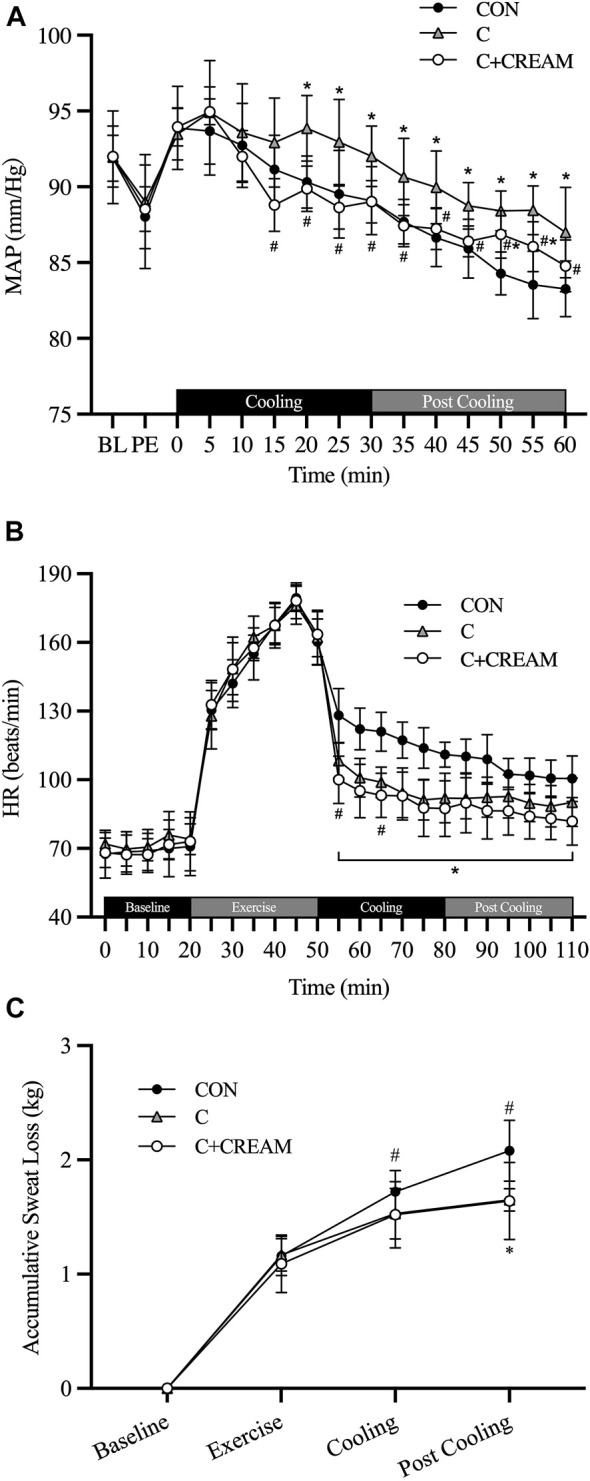
Mean arterial pressure (MAP), heart rate (HR), and accumulative sweat loss. Changes in MAP **(A)** during cooling and post cooling, HR **(B)** and accumulative sweat loss **(C)** during baseline, exercise, cooling, and post cooling are presented. BL, baseline; PE, post exercise; CON, control cooling; C, ice-suit cooling; C + CREAM, ice-suit cooling with cutaneous application of analgesic cream. Two-way repeated measures ANOVA with a Tukey’s post-hoc test for all. Data are presented as the mean ± SD (*n* = 10). *p < 0.05 vs. CON. ^#^ *p* < 0.05 vs. C.

An increase in blood pressure occurs during body cooling primarily due to sympathetically mediated peripheral vasoconstriction (Koehn et al., 2020). The elevated blood pressure during cooling implies a risk of cardiovascular side effects in response to cooling (Koehn et al., 2012). Indeed, cooling interventions such as CWI are occasionally reported as inducing an increase in blood pressure (Tipton et al., 2017), although the majority of research regarding cooling intervention for hyperthermia have reported no such changes. In our study, an elevated MAP was seen during the 30-min ice-suit cooling period relative to control recovery (Figure 5A). Interestingly, cooling with analgesic cream showed an attenuated MAP response which was comparable to the control recovery (Figure 5A). Methyl salicylate, a major ingredient of topical analgesic cream used in the current investigation, is known to induce an analgetic effect due to desensitization of TRPV1 on sensory neurons after initial activation (Ohta et al., 2009). It is possible that cold-induced activation of sensory afferent neurons, at least in part, may have been attenuated by TRPV1 desensitization induced by methyl salicylate applied *via* analgesic cream, which blunted reflex vasoconstriction and consequently decreased the MAP response during cooling. Another hypothesis is that the L-menthol in the analgesic cream used in the current study may reach systemic circulation and suppress RhoA/ROCK pathway in response to skin cooling, as has been previously observed in humans (Martin et al., 2004). In this context, we note that several studies have previously found that oral supplementation with menthol lowers blood pressure in hypertensive animals (Sun et al., 2014; Xiong et al., 2017), and that sympathetic nervous activity may be inhibited by the methyl salicylate-induced activation of TRPV1 (Ohta et al., 2009; Alawi et al., 2015).

Recently, Keen and Miller criticized the use of cooling garments in serious cases of hyperthermia such as exertional heat stroke (EHS), and concluded that cooling vests are ineffective in rapidly reducing T_core_ relative to CWI (Vest 0.03∼0.053 vs. CWI ∼0.2°C/min) (Keen and Miller, 2017). In our experiment, the cooling rate observed with the ice-suit application was 0.036°C ± 0.005°C/min during the 30-min cooling time, which is consistent with the data reported by Keen and Miller. Cooling with CWI, however, normally lasts less than 10 min (Nye et al., 2016), while cooling with an ice-vest can last from anywhere between 10 and 30 min (Keen and Miller, 2017). Our data shows that cooling during the first 10 min occurred at a rate of 0.048°C ± 0.011°C/min in the ice-suit cooling condition and a rate of 0.067°C ± 0.018°C/min in the condition with an ice-suit an analgesic cream (Figure 4C). These cooling rates decreased over the course of the next 20 min (Figure 4C) as the hyperthermic bodies were progressively cooled. Given that an acceptable cooling rate for hyperthermia is considered >0.08°C/min (Keen and Miller, 2017), the cooling rate observed in our C + CREAM group does not seem to be satisfactory. Our data, however, does leave open the possibility that ice-suit cooling, with some modifications to cooling area and cooling modality, remains a viable cooling (e.g., CWI).

Some previous studies have reported no significant differences in cooling on the core body between ice-suit and control recovery (i.e., no intervention) tests (Lopez et al., 2008; Brade et al., 2010; Hostler et al., 2010; DeMartini et al., 2011). We showed that the cooling effect of an ice-suit was significantly greater than that of passive recovery (Figure 4A). This discrepancy can be explained by the fact that environmental temperatures during recovery were thermoneutral (∼24°C) in previously conducted experiments (Lopez et al., 2008; Brade et al., 2010; Hostler et al., 2010; DeMartini et al., 2011) while room temperature during recovery in our experiments was maintained at ∼35°C. Based on our observations, an ice-suit seems to be an effective cooling strategy for treating hyperthermia in a hot environment, but ineffective when recovery is being performed in a comfortable environment.

We also assessed physiological parameters including T_core_, MAP, HR, and accumulative sweat loss (Figures 4, 5) after cooling interventions (Post Cooling) to determine whether heat gain had increased in individuals to whom the analgesic cream had been applied, as a result of any sustained cutaneous vasodilation in a hot environment (35°C). However, no differences were observed between conditions during Post Cooling (Figures 4, 5).

### 4.1 Limitations

There are some limitations to the current study. Although a potent vasodilatory effect of analgesic cream during local skin cooling was observed in Study 1, the heat loss effects of analgesic cream applied during whole-body cooling in exercise-induced hyperthermia do not seem to be physiologically meaningful (0.1°C∼0.2°C difference in each time point). Unlike local cooling-induced vasoconstriction in the skin, a whole-body cooling in this investigation is thought to elicit reflex cutaneous vasoconstriction, which may have decreased the effectiveness of analgesic cream application. Also, We note that the ice-suit used in the current study left a large amount of body surface area uncovered, as it enclosed only the torso (back, chest, shoulders, and abdomen) and legs (anterior thigh and knee regions and posterior lower legs). The existence of uncovered body areas, including the shoulders, arms, and neck, would have limited any cooling effects, particularly in the current study in which cooling recovery occurred in a hot environment. To maximize the cooling effect of the applied analgesic cream, cooling areas or modalities need to be carefully considered in future investigations.

Gel-filled packs were utilized in the ice-suit in the current study. The temperature of the gels was maintained at 7°C when frozen but the cooling effect of the gel was attenuated as frozen gels are continuously defrosted in a hot environment. While gel packs were replaced after 15 min of cooling in the current study (Figure 1), the replacement of gel packs may not be possible in clinical settings. The gel mixture or ice water that circulates through the suit should be optimized to augment the cooling effects of an ice-suit.

Also, we excluded females in the current study because the magnitude of change in T_core_ can be affected by subcutaneous fat level of different sexes (Boehm and Miller, 2019). Therefore, interpretation of the current study should be limited to males and future studies that include both males and females are needed to generalize the effect of analgesic cream application during skin cooling.

The molecular mechanisms underlying attenuated cold-induced vasoconstriction in the cutaneous microvasculature were not investigated in the current study. Our interpretation of our findings concerning augmented heat loss during cooling *via* cutaneous application of analgesic cream containing methyl salicylate and L-menthol are therefore limited. Importantly, further data regarding ROCK and TRPV1 pathways during skin cooling with analgesic cream application will be needed to understand the observed effects on heat loss in exercise-induced hyperthermia. Additional measurements of autonomic regulation and oxygen consumption could have, at least in part, explained the current findings.

## 5 Perspectives

Skin cooling is a widely utilized technique to decrease core body temperature in response to hyperthermia. Cutaneous vasoconstriction in response to external cold stimulation can restrict the capacity of this treatment, as a cold-induced reduction in skin blood flow may limit heat transfer between the cooled skin and core body. In this study, we showed that cutaneous application of OTC analgesic cream containing methyl salicylate and L-menthol can partially counteract cold-induced vasoconstriction in the skin during cooling. We also revealed that core body heat loss could be accelerated by application of an ice suit in conjunction with cutaneous application of analgesic cream in exercise-induced hyperthermia. These OTC topical analgesics have been utilized for the treatment of muscle and joint pain for decades; however, to date no research has focused on their vasodilatory functions in the skin. Given their established safety profiles and affordability, topical cutaneous analgesics containing vasodilatory agents may be a useful tool of boosting the cooling effects of an ice-suit in situations where rapid cooling is called for.

## Data Availability

The original contributions presented in the study are included in the article/supplementary material, further inquiries can be directed to the corresponding author.
